# Exploring the association between lifetime traumatic experiences and positive psychotic symptoms in a group of long-stay patients with schizophrenia: the mediating effect of depression, anxiety, and distress

**DOI:** 10.1186/s12888-023-04531-3

**Published:** 2023-01-12

**Authors:** Clara Rahme, Nisreen El Kadri, Chadia Haddad, Feten Fekih-Romdhane, Sahar Obeid, Souheil Hallit

**Affiliations:** 1 Research Department, Psychiatric Hospital of the Cross, Jal Eddib, Lebanon; 2 Faculty of Science, Lebanese University, Fanar, Lebanon; 3 INSPECT-LB: National Institute of Public Health, Clinical Epidemiology and Toxicology, Beirut, Lebanon; 4 Modern University of Business Sciences, Damour, Lebanon; 5 The Tunisian Center of Early Intervention in Psychosis, Department of psychiatry “Ibn Omrane”, Razi hospital, 2010 Manouba, Tunisia; 6 Tunis El Manar University, Faculty of Medicine of Tunis, Tunis, Tunisia; 7 Social and Education Sciences Department, School of Arts and Sciences, Lebanese American University, Jbeil, Lebanon; 8 School of Medicine and Medical Sciences, Holy Spirit University of Kaslik, P.O. Box 446, Jounieh, Lebanon; 9 Applied Science Research Center, Applied Science Private University, Amman, Jordan

**Keywords:** Trauma, Positive psychotic symptoms, Schizophrenia, Anxiety, Depression, Distress

## Abstract

**Background:**

Positive psychotic symptoms of schizophrenia are generally characterized by hallucinations and delusions. We propose to assess the relationship between total composite trauma and positive psychotic symptoms, along with the mediation effect of cognition, fear of COVID-19, insomnia, anxiety, distress, and depression of Lebanese patients with schizophrenia.

**Methods:**

A cross-sectional study was carried out, between June and July 2021, by deriving data from 155 long-stay in-patients diagnosed with schizophrenia.

**Results:**

Depression, anxiety, and distress but not cognitive impairment, insomnia, and fear of COVID-19) mediated the association between lifetime traumatic experiences and positive psychotic symptoms. Higher traumatic experiences were associated with greater depression, anxiety, and distress, indicating a significant positive total effect on positive psychotic scores. Moreover, higher depression, anxiety, and distress were significantly associated with higher positive psychotic symptoms.

**Conclusion:**

Our results contribute to the existing knowledge by suggesting other possible intervention paths through mediating factors. Interventions that improve anxiety, depression, and distress severity may be effective in reducing positive psychotic symptoms among patients with schizophrenia having experienced lifetime trauma.

## Background

Schizophrenia is a severe psychiatric disorder with complex cognitive and behavioral symptoms caused by genetic and/or environmental factors affecting certain brain areas and circuits, resulting in disrupted brain development [1]. It is characterized by symptoms of delusions, hallucinations, disorganized speech, disorganized behavior, and negative symptoms [2, 3]. Negative symptoms refer to a diminution or absence of normal behaviors related to motivation and interest or expression [4]. According to the World Health Organization (WHO), approximately 24 million people, or 1 in 300 persons, are affected with schizophrenia globally [5]. Schizophrenia is particularly prevalent in young adults between 20 and 30 years of age and leads to disability in about half of the patients [3].

Several propositions have been made for the mechanisms by which cumulative adversity confers psychosis risk, including the suggestion that exposures create vulnerability to psychotic experience through toxic effects on biological, cognitive, and affective systems, which in turn may be amplified by additive environmental stressors over time [6]. Schizophrenia has a multifactorial etiology, with complex gene–environment interactions leading to the emergence and progression of psychotic symptoms [7]. In particular, early-life environmental risk factors have proven to play a major role in altering neurodevelopmental trajectories, and in turn, contributing to the development of prodromal symptoms in predisposed individuals [8]. One of the environmental factors is trauma experiences across the lifespan, especially during childhood and adolescence [9, 10]. A previous meta-analysis found that traumas in childhood may lead to hallucinations and delusions within psychotic disorders [11]. This meta-analysis revealed that among individuals with psychosis, childhood trauma was significantly correlated with the severity of hallucinations and delusions [11].

### The relationship between traumatic experiences and positive psychotic symptoms

Stressful or traumatic events experienced in childhood or adolescence have a lifelong impact on mental and physical health [12]. Exposure to traumatic events is associated with posttraumatic stress and other common childhood emotional and behavioral problems. Extensive literature links childhood trauma, particularly maltreatment, to adult psychopathology and impairment [13]. Childhood trauma can be assumed to be a severe form of stress that renders individuals more vulnerable to developing schizophrenia [3]. Patients with schizophrenia have been shown to experience a higher prevalence of traumatic experiences than patients with the affective disorder [14] and healthy individuals from the general population [15]. There is strong evidence from a meta-analysis of patient-control, prospective, cross-section,al and cohort studies that exposure to traumatic events and experiences is associated with an increased risk for psychosis in adulthood [16]. Beyond being predictive of the later development of schizophrenia in healthy individuals [9, 10], being exposed to trauma has also been associated with poorer physical and mental health and functional disability in the general population [17] and patients with schizophrenia [18, 19]. Morrison et al. have explored the association between psychosis and trauma [20]. They found that there was a very high prevalence of exposure to traumatic life events in people with psychosis [20]. In addition, the severity of trauma was associated with the severity of both PTSD and psychotic symptoms in people with psychosis [20]. Despite these data, the nature and mechanisms underlying the relationship between trauma exposure and psychotic symptoms need further investigation [21]. Also, traumatic experiences have been linked to various mental disorders such as depression [22], bipolar disorders [23], eating disorders [24], borderline personality disorder [25], and substance-related disorders [26]. Trauma is thus far from being specifically involved in the etiology of psychotic disorders, which further complicates our understanding of the pathways leading from trauma to psychosis. Hence, a strong need for further exploring the relationship and possible mediators between traumatic experiences and psychotic symptoms in patients with schizophrenia.

### Potential mediators on the association between lifetime traumatic experiences and positive psychotic symptoms

**Fig. 1:**
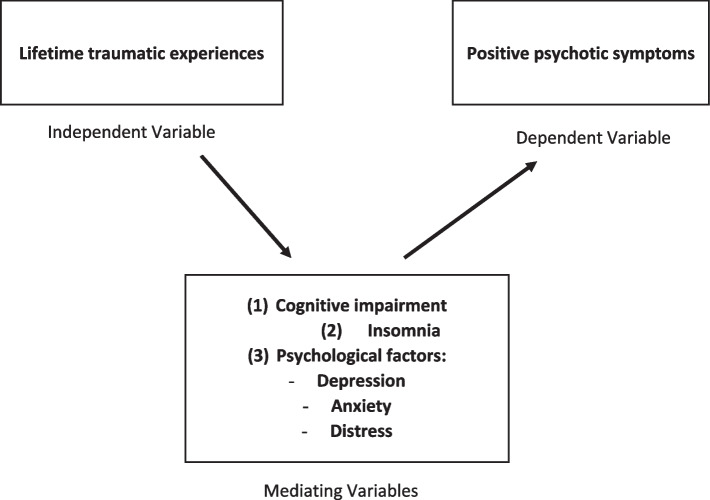
Conceptual framework of the association between lifetime traumatic experiences and positive psychosis symptoms, while considering cognitive impairment, insomnia, and psychological factors as mediators [27]

To better understand the interplay between lifetime traumatic experiences and positive psychotic symptoms, we theoretically hypothesized three sets of variables as mediators in this relationship: (1) cognitive impairment, (2) insomnia, and (3) psychological factors (i.e., depression, anxiety, distress, and fear of COVID-19) as shown in fig. 1.

The choice of the cognitive factor was based on Morrison’s integrative cognitive approach, which suggests that experiencing trauma at any age can alter a child’s or adult’s attributional style, fostering negative beliefs about the self, the world, and others [28]. In the wake of trauma, adolescents often consider themselves vulnerable, others untrustworthy, and the world as dangerous and unsafe. These negative belief structures can alter attributional styles, making paranoid, distressing interpretations of ambiguous events more likely [10]. Up to 75% of people with schizophrenia have significant cognitive deficits which are often the first signs of schizophrenia [29]. In general, schizophrenia patients experience impairments in a wide range of cognitive processes, including memory, attention, motor skills, executive function, and social cognition [30]. Cognitive impairment is highly prevalent in schizophrenia, possibly because both psychotic symptoms and impaired cognition share similar etiology – genetic and/or environmental features [31]. Accordingly, physical or emotional trauma during childhood may cause cognitive impairment and increase the risk of developing schizophrenia or other mental disorders later in life [32]. Childhood maltreatment and neglect are known to have a detrimental impact on the cognitive functioning of patients with schizophrenia and bipolar disorder [33]. Childhood neglect was a predictor of impaired social cognition and poorer verbal learning in patients with first-episode schizophrenia [34]. At the same time, cognitive impairment (e.g., large errors in memory) has been found to be associated with higher levels of positive symptom s[35].

Based on literature data, we also propose the hypothesis that insomnia may represent a mediator underlying the relationship between trauma experiences and positive psychotic symptoms. Insomnia is common in patients with schizophrenia and has been suggested as a risk factor for psychotic symptoms emergence and exacerbation [36, 37]. Research found that the presence of sleep disturbance, especially clinically significant insomnia, likely worsens clinical outcomes for patients with schizophrenia [38]. Evidence also supported that psychotic symptoms and experiences tend to exacerbate as a result of sleep disturbances in clinical [36, 39] and non-clinical populations [40]. Sleep is generally impacted both immediately following a trauma and in the long-term with affected sleep domains including nightmares, fragmented sleep, initial insomnia, fatigue, sensations at night, light sleep, and night anxiety. These effects on normal sleep processes are considered one of the most frequent and distressing complaints following a traumatic event [41]. In addition, longitudinal studies indicate that sleep disruption, before and following trauma exposure leads to exacerbation of subsequent trauma-related distress, specifically for those with pre-existing insomnia [42].

In terms of psychological variables, our choice of fear of COVID-19 as a mediator was motivated by the increasing amount of evidence that the pandemic substantially affected people with schizophrenia and has even been shown to contribute to the emergence of de novo psychotic symptoms [43]. Patients with schizophrenia are postulated to be at a higher risk of acquiring COVID-19 and having a poorer health outcome and this can be due to the symptoms of this disease as delusions, hallucinations, disorganized behavior, cognitive impairment, and impaired insight [44, 45]. Moreover, increased attention has been given to psychotic symptoms as they occur in patients infected with COVID-19 without any psychiatric disorders, putting patients with schizophrenia at high risk [46]. At the same time, individuals who experienced childhood trauma have been demonstrated to exhibit high levels of COVID-19-related fear [47].

On the other hand, psychological distress can also mediate the association between trauma and psychosis in schizophrenia. There is sufficient evidence that traumatic experiences are linked to the development of depression, anxiety, and distress later in life [48–50]. Hartley’s systematic review explored the influence of anxiety and depression on positive psychotic symptoms and if those variables were the cause of the emergence and persistence of the psychosis. They found that both anxiety and depression are associated in meaningful ways with the severity of delusions and hallucinations, the distress they elicit, and their content [51]. The cross-sectional nature of the majority of studies and the focus on certain symptom subtypes couldn’t reveal the causality of this association between anxiety, depression, and psychosis. However, the findings of this systemic review may imply that anxiety and depression could be targets for therapeutic intervention [51].

Findings relevant to the severity of psychotic experiences have demonstrated that anxiety is related to levels of paranoia, and delusions and can trigger acute augmentation in auditory hallucinations [26]. Another research showed that the severity of positive psychotic symptoms is significantly associated with increased severity of anxiety symptoms [52]. Moreover, depression was significantly associated with symptom severity in both chronic and early psychosis groups [26]. Mood instability and affective dysregulation are highly prevalent in patients with schizophrenia and have been suggested to give rise to psychotic symptoms [53].

### The present study

Schizophrenia remains a chronic debilitating disorder, and its management can be challenging. Positive symptoms of schizophrenia can be a result of many factors that could worsen the outcome and quality of life of patients [54]. Establishing the role of lifetime traumatic experiences in psychotic disorders is the first step in anticipating and eventually mitigating the adulthood development of these life-altering disorders. Not to forget the role of culture in severe mental illnesses such as schizophrenia which requires adequate attention and continued research [55]. An anterior Lebanese study aimed to delineate psychotic symptoms with sexual content, along with their relationships with the severity of schizophrenia symptoms and childhood abusive events [56]. They found that 36.5 and 50.3% of the participants screened positive for current and lifetime episodes of sexual delusions and/or hallucinations, respectively [56]. Another cross-sectional study examined a sample of Lebanese patients with schizophrenia to identify clinical risk factors for aggressiveness including child abuse. They found that higher physical and sexual abuse, alcohol drinking, having a history of head trauma, and male gender were significantly associated with higher mean aggression scores [57]. Thus, studying the factors that may affect positive symptoms of schizophrenia among the Lebanese population is crucial to provide better ways of treatment. Therefore, our principal objective was to assess the relationship between lifetime traumatic experiences and positive psychotic symptoms, along with the mediation effect of cognitive impairment, insomnia, and psychological factors among Lebanese patients with schizophrenia.

## Methods

### Study design

A cross-sectional study was carried out, between June and July 2021, by deriving data from 155 in-patients diagnosed with schizophrenia who were on a long stay from the Psychiatric Hospital of the Cross (PHC). Each patient had the right to accept or refuse to participate in the study if eligible; those who agreed to enroll received no financial rewards in return.

### Participants

**Fig. 2:**
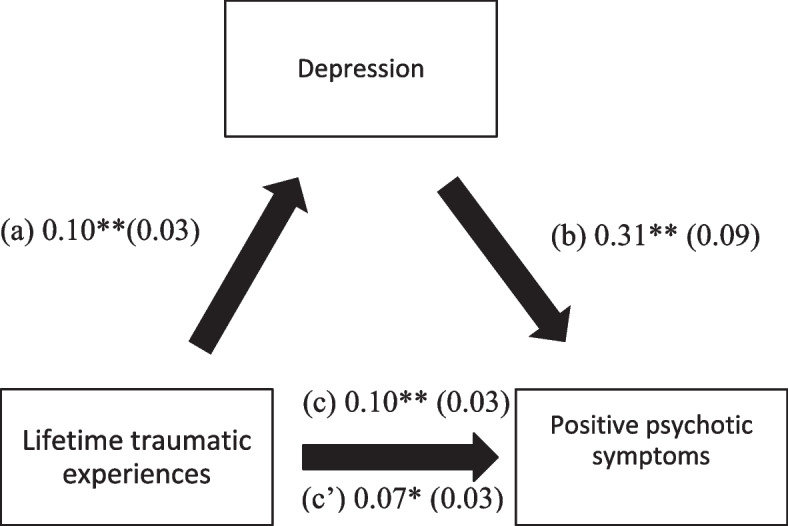
(**a**) Relation between lifetime traumatic experiences and depression (R^2^ = 20.40%); (**b**) Relation between depression and positive PANSS (R^2^ = 20.80%); (**c**) Total effect of the relation between lifetime traumatic experiences and positive PANSS (R^2^ = 20.8%); (c’) Direct effect of the relation between lifetime traumatic experiences and positive PANSS. Numbers are displayed as regression coefficients (standard error). **p < 0.01; *p < 0.05

155 in patients were recruited for this study (101 males and 54 females). Inclusion criteria for participants were as follows: (i) a diagnosis of schizophrenia spectrum according to the DSM-5 criteria, as confirmed by two independent psychiatrists of PHC, (ii) being chronic inpatients with a minimum age of 18, (iii) being able to recognize the aim of the current study and being aware of their approval to participate (in case of inability to consent a family member did). All data concerning patients’ exclusion is represented in Fig. 2.

### Ethical approval

The Psychiatric Hospital of the Cross Ethics and Research Committee approved this study protocol (HPC-016-2021). Informed consent was collected from all participants.

### Minimal sample size calculation

The G-power software estimated a minimal sample of 139 patients based on an R^2^ value of 0.13, a 5% risk of error, 80% power, and 15 variables to be entered in the multivariable analysis model [58].

### Materials

The battery of questionnaires used was in Arabic, administered by a researcher well trained to use the scales, and needed around 60 min to be filled; each questionnaire consisted of two parts:

The first part was to collect participants’ sociodemographic characteristics; it collected information about age, gender, education, and marital status. Information concerning patients was collected from the PHC records, such as the age of onset of illness, medication taken and its equivalent chlorpromazine dose (defined as a dose of antipsychotic comparable to 100 mg of CPZ), personal history of suicide, and family history of psychiatric diseases. The second part included the following scales:

#### Montreal cognitive assessment scale (MOCA)

The MOCA, validated in Arabic [59], is a one-page 30 -point screening test designed to detect mild cognitive impairment (MCI). It is a screening tool for cognitive impairment, which assesses eight cognitive domains (using 14 questions) including attention, concentration, memory, visuospatial skills, executive functions, abstraction, language, orientation, and calculation. It has a good internal consistency in this study (Cronbach’s alpha = 0.915) [60]. The participant is given a grade for each question depending on the number of right answers per question. MOCA has a minimum score of 0 and a maximum score of 30. A total score below 26 indicates mild cognitive impairment. If years of education are less than 12, one point is added to the total score [61].

#### Traumatic antecedents’ questionnaire (TAQ)

Childhood traumatization experiences were assessed by the Traumatic Antecedents Questionnaire (TAQ) developed by van der Kolk. The 42-item questionnaire gathered information about the frequency and severity of traumatic and adaptive experiences [62] in 10 different subscales. The 2 subscales measuring adaptive experiences cover safety and competence, while the other 8 that measure traumatization are neglect, separation, emotional abuse, physical abuse, sexual abuse, witnessing, other trauma, and alcohol and drugs [62, 63]. All of these adverse experiences required a response for each one of the four developmental age groups: early childhood (0-6 years), latency (7- 12 years), adolescence (13-18 years), and adulthood (over 18 years), rating the extent to which each statement describes their experience on a scale from 0 to 3 where “0” means “never or not at all” and “3” means “often or very much”. Participants can also choose “Don’t Know” which will be considered as missing information during scoring. The TAQ allows the calculation of composite scores for each of the 10 domains, as well as for the four developmental periods of each domain. The total TAQ scores, assessing all forms of trauma during the lifetime (the four developmental periods) were considered in the present study. The Arabic version of the TAQ was translated by the forward-backward method. A healthcare professional translated it into Arabic language from the original English one, and a professional psychologist performed the backward translation into English language. The process was supervised and compared by the study supervisor. The Cronbach’s alpha of this scale in this study was 0.895.

#### Positive and negative syndrome scale (PANSS)

The Positive and Negative Scale [64], validated in Arabic [65], was used to assess the severity of symptoms for schizophrenia, composed of a 7-item subscale measuring positive symptoms covering delusions, conceptual organization, hallucinations, excitement, grandiosity, suspiciousness/persecution, and hostility, in addition to 7-item subscale measuring negative symptoms assessing blunted affect, emotional withdrawal, poor rapport, passive social withdrawal, difficulty in abstract thinking, lack of spontaneity and flow of conversation, and stereotyped thinking. Additionally, a 16-item subscale measuring general psychopathology symptoms assessing Somatic concern, Anxiety, Guilt feelings, Tension, Mannerisms and posturing, Depression, Motor retardation, Uncooperativeness, Unusual thought content, Disorientation, Poor attention, Lack of judgment and insight, Disturbance of volition, Poor impulse control, Preoccupation, and Active social avoidance. The 30 items all together were scored on a 7-point Likert scale with 1 (absent) as the lowest score and 7 (extreme) as the highest one (Cronbach’s alpha =0.961) The administered Arabic version in this study is validated in Lebanon [65]. In this study, we only used the positive symptoms sub-scale.

#### Lebanese anxiety scale

The 10-item scale validated among adults [66] and adolescents [67], is used to screen for anxiety in the general population. Questions 1 to 7 are scored on a 5-point Likert scale from 0 (not present) to 4 (very severe), while items 8–10 are graded on a 4-point Likert scale from 1 (never/almost never) to 4 (almost always). Higher scores indicate higher anxiety.

#### Lebanese insomnia scale (LIS-18)

The Lebanese Insomnia Scale [68], Includes 18 elements evaluating insomnia and its underlying causes. Both answers are assessed using the 1 to 5 Likert scale. Low scores represent more severe sleep disruptions. Cronbach’s alpha was 0.821.

#### Hamilton depression rating scale

The Hamilton Depression Rating Scale (HAM-D), validated in Arabic [69], was used to assign the severity of depression by rating several factors including anxiety, suicide ideation, mood, guilt feeling, agitation, or psychomotor retardation, somatic symptoms, insomnia, and concerning weight loss. It consists of 17 items with Likert scale of either 0 to 4 or 0 to 2. The final score is obtained after adding the scores of each of the 17-item scale, where 0 to 7 was considered as a normal state, 8 to 13 as mild depression, 14 to 18 as moderate depression, 19 to 22 as severe depression, and greater than or equal 23 as very severe depression. (Cronbach’s alpha in this study = 0.637).

#### Beirut distress scale (BDS-10)

Beirut Distress Scale (BDS-10) is used to assess mental and psychological distress with a total of 10 items exclusively related to stress, on a 4-point Likert scale starting from 0 (never) to 3 (very much) with higher scores indicating higher stress. It is an abbreviated version of the BDS-22 with a Cronbach’s alpha of 0.954 [70].

#### Fear of COVID-19 scale

The Fear of COVID-19 Scale, a seven-item scale, has robust psychometric properties. Previously validated [71] in Arabic, it is reliable and valid in assessing fear of COVID-19 among the general population and will also be useful in allaying COVID-19 fears among individuals [72]. The scale consists of 7 items evaluated on a 5-point Likert scale, with 1 (strongly disagree) and 5 (strongly agree). The total score is the sum of the scores of the 7 items, with a minimum score of 7 and a maximum of 35, where a higher score indicates greater fear of COVID-19. The validated Arabic version of this scale [71] was administered to our participants with a Cronbach’s value of 0.88.

### Statistical analysis

Data analysis was conducted using SPSS software version 25. The sample was normally distributed as verified by the skewness and kurtosis of the positive PANSS score, which varied between − 2 and + 2 (George, 2011). The Student t and ANOVA tests were used to compare two and three or more means respectively. The Pearson correlation test was used to correlate two continuous variables. The PROCESS SPSS Macro version 3.4, model four [73] was used to calculate all pathways (Pathway A from the independent variable to the mediator, Pathway B from the mediator to the dependent variable, and Pathway C from the independent to the dependent variable). Pathway AB calculated the indirect effect; the latter was deemed significant when the macro-generated bias-corrected bootstrapped 95% confidence intervals (CI) did not pass by zero. The model was adjusted over covariates that showed a *p* < 0.25 in the bivariate analysis. Significance was set at a *p* < 0.05.

## Results

### Sociodemographic characteristics

The sample consisted of 155 participants, with a mean age of 56.68 ± 10.47 years and 65.2% males. Other characteristics and descriptions of the scores can be found in Table 1.

**Table 1:** Sociodemographic characteristics of the participants (*N* = 155)

Variable	N (%)
**Gender**	
Male	101 (65.2%)
Female	54 (34.8%)
**Marital status**	
Single/divorced/widowed	22 (14.2%)
Married	133 (85.8%)
**Education level**	
Illiterate	18 (11.7%)
Primary	39 (25.3%)
Complementary	49 (31.8%)
Secondary	33 (21.4%)
University	15 (9.7%)
**Infected by the coronavirus**	
No	33 (21.3%)
Yes	122 (78.7%)
**Family history of psychiatric diseases**	
No	94 (60.6%)
Yes	61 (39.4%)
	**Mean ± SD**
Age (in years)	56.68 ± 10.47
Chlorpromazine equivalent dose (mg)	1189.81 ± 2299.35
Cognitive function (MOCA score)	13.94 ± 6.43
PANSS positive score	16.88 ± 7.18
Depression	10.69 ± 6.41
Anxiety	14.48 ± 8.77
Distress	9.79 ± 6.91
Insomnia	36.16 ± 15.09
Fear of COVID-19	18.34 ± 6.45
Lifetime traumatic experiences	38.59 ± 15.54

### Bivariate analysis

The results of the bivariate analysis are displayed in Tables 2 and 3. Higher positive PANSS scores were significantly associated with more depression, anxiety, distress, insomnia, and Lifetime traumatic experiences.

**Table 2:** Correlation between the positive PANSS score and continuous variables

Variable	r	p
Age	0.07	0.363
Age of onset of the disease	−0.11	0.150
Number of hospitalizations	0.10	0.221
Depression	0.36	**< 0.001**
Anxiety	0.30	**< 0.001**
Distress	0.37	**< 0.001**
Insomnia	0.16	**0.049**
Fear of COVID-19	0.04	0.644
Lifetime traumatic experiences	0.26	**0.001**

Numbers in bold indicate significant *p*-values; r = Pearson correlation coefficient

**Table 3:** Bivariate analysis of the positive PANSS score and categorical variables

Variable	Mean ± SD	p
**Gender**		0.130
Male	16.28 ± 6.54	
Female	18.09 ± 8.25	
**Marital status**		0.354
Single/divorced/widowed	15.63 ± 6.62	
Married	17.10 ± 7.27	
**Education level**		0.929
Illiterate	16.28 ± 6.65	
Primary	16.44 ± 7.28	
Complementary	17.56 ± 7.70	
Secondary	17.12 ± 7.76	
University	16.31 ± 4.87	
**Infected by the coronavirus**		0.072
No	14.91 ± 5.39	
Yes	17.48 ± 7.67	
**Family history of psychiatric diseases**		0.377
No	16.43 ± 5.48	
Yes	17.55 ± 9.13	

Numbers in bold indicate significant p-values

### Mediation analysis

**Fig. 3:**
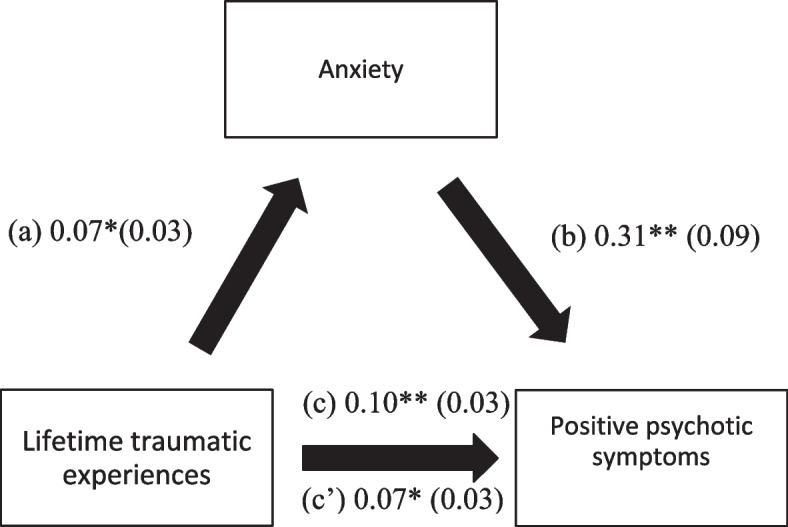
(**a**) Relation between lifetime traumatic experiences and anxiety (R^2^ = 20.40%); (**b**) Relation between anxiety and positive PANSS (R^2^ = 17.70%); (**c**) Total effect of the relation between lifetime traumatic experiences and positive PANSS (R^2^ = 14.80%); (c’) Direct effect of the relation between lifetime traumatic experiences and positive PANSS. Numbers are displayed as regression coefficients (standard error). **p < 0.01; *p < 0.05

**Fig. 4:**
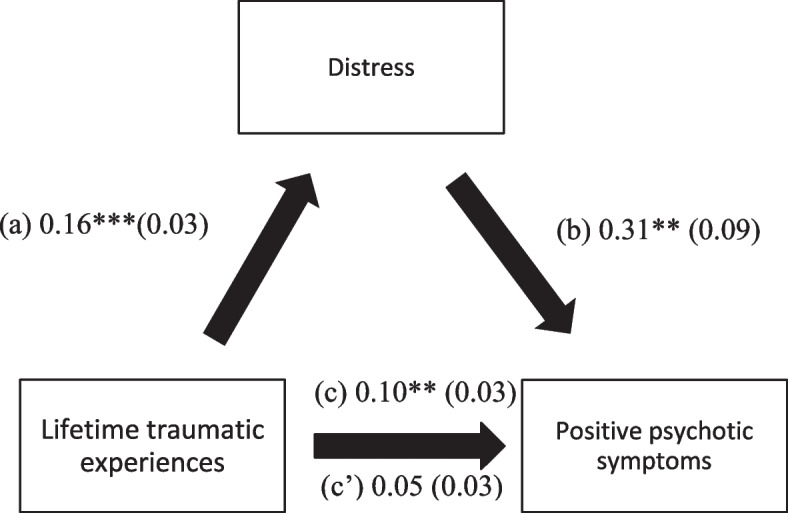
(**a**) Relation between lifetime traumatic experiences and distress (R^2^ = 27.80%); (**b**) Relation between distress and positive PANSS (R^2^ = 21.10%); (**c**) Total effect of the relation between lifetime traumatic experiences and positive PANSS (R^2^ = 14.80%); (c’) Direct effect of the relation between lifetime traumatic experiences and positive PANSS. Numbers are displayed as regression coefficients (standard error). ***p < 0.001; **p < 0.01; *p < 0.05

Depression, anxiety, and distress (but not cognitive impairment, insomnia, and fear of COVID-19) mediated the association between traumatic experiences and positive PANSS symptoms (Table 4). Higher lifetime traumatic experiences were associated with greater depression, anxiety, and distress and a significant positive total effect on positive PANSS scores. Moreover, higher depression, anxiety, and distress were significantly associated with higher positive psychotic symptoms (Figs. 2, 3, and 4).

**Table 4:** Mediation analyses results, taking lifetime traumatic experiences as the independent variable, and the positive PANSS score as the dependent variable

Mediator	Direct effect			Indirect effect		
	Beta	SE	p	Beta	Boot SE	Boot CI
**Cognitive function**	0.10	0.03	0.003	0.004	0.01	−0.01; 0.02
**Depression**	0.07	0.03	0.030	0.03	0.02	0.01; 0.07*
**Anxiety**	0.07	0.03	0.029	0.03	0.02	0.003; 0.06*
**Stress**	0.05	0.03	0.132	0.05	0.02	0.02; 0.09*
**Insomnia**	0.09	0.03	0.005	0.01	0.01	−0.01; 0.02
**Fear of COVID-19**	0.10	0.03	0.002	0.001	0.003	−0.01; 0.01

*indicates significant mediation model; all models were adjusted over gender, age of onset of the disease, number of hospitalizations, and infection by COVID-19

## Discussion

The objectives of this study were to assess the relationship between lifetime traumatic experiences and positive psychotic symptoms, along with the mediation effect of cognitive impairment, insomnia, and psychological factors among Lebanese long-stay patients with schizophrenia. Our findings revealed a significant positive effect of lifetime traumatic experiences on positive psychotic symptoms. In addition, the results of the mediation analysis showed that depression, anxiety, and distress (but not cognitive impairment, insomnia, and fear of COVID-19), had a significant indirect effect on the association between traumatic experiences and positive psychotic symptoms.

As for the direct effect, we extend previous research by showing that higher traumatic experiences were associated with higher levels of depression, anxiety, and distress. High rates of childhood trauma are reported in schizophrenia and are thought to be important in the genesis of the disorder [74]. Childhood trauma is an event or a series of stressful events that renders individuals more vulnerable to developing schizophrenia. Previous study showed that trauma exposure and perceived stress also predicted higher depression scores [75]. Additionally, a strong positive correlation was found between childhood maltreatment and psychotic symptoms [76]. Higher levels of childhood trauma were correlated with higher levels of attenuated positive symptoms [3]. An anterior study linked early trauma to (positive) psychotic symptoms in first-episode schizophrenia and demonstrate a dose–response relationship between childhood trauma and psychotic symptoms in cases with chronic psychosis at New York Psychiatric Institute [75]. Another investigation in the Southern China population observed a dose-response relationship between the severity of childhood trauma and the incidence of psychotic experiences and reported that cessation of childhood trauma decreased the chance of an episode of psychosis [76].

Depression, anxiety, and distress mediated the association between total composite trauma and positive PANSS symptoms. This is consistent with a previous study revealing that mood instability (distress, anxiety, and depression) may act as a mediator between traumatic experiences such as bullying and persecutory ideation, as well as childhood sexual abuse and psychosis [74]. Cross-sectional studies have demonstrated that negative perceptions of the self, anxiety, and depression partially mediated associations between trauma (not always limited to childhood) and psychotic symptoms [77–79]. A possible explanation for this mediation is that childhood trauma may initially give rise to affective symptoms, and only later to psychotic symptoms, which has been referred to as ‘the affective pathway to psychosis’ [80]. This was confirmed by the results of anterior research in which it was consistently found that patients with a history of childhood trauma were more likely to have a combination of multiple symptom domains compared to their non-traumatized counterparts. Importantly, childhood trauma was associated with multiple symptom clusters rather than with isolated symptoms. The authors concluded that, instead of increasing the risk for a specific disorder, childhood trauma may increase the risk for stress-related disorders through changes in the hypothalamic-pituitary-adrenal axis, alterations of which have been reported in several mental disorders including psychosis [81].

Furthermore, we did not find a significant mediating effect for insomnia, fear of COVID, and cognition in the association between traumatic experiences and positive psychotic symptoms. Research working on how developmental trauma induces vulnerability to psychotic symptoms did not find a mediating effect on cognition which was consistent with our study [82]. Insomnia was significantly associated with previous trauma experience [83] and was also significantly associated with higher psychosis [84]. However, no study to date has examined all three variables together. The sample applies to the fear of COVID-19 variable. Previous studies observed a positive association between fear of COVID-19 and psychosis [85] and trauma was assessed only after COVID infection [86]. A possible explanation for this inconsistency is that the patients in this study are in long stay in the hospital, which can make insomnia and fear of COVID-19 more controlled and less impactful.

The findings of this study present several clinical implications. Our findings provide more support to the direct effect of lifetime traumatic experiences on positive psychotic symptoms, suggesting a need to consider these experiences when aiming at improving symptoms in schizophrenia. Several therapies may be indicated in patients with schizophrenia having a concomitant history of trauma, such as psychologically oriented trauma-based therapies [87], cognitive-oriented trauma-focused therapies [9], and therapies such as mindfulness [88]. Such therapies have proven to be well tolerated and effective in addressing both trauma symptoms and psychotic symptoms [89]. In addition, our results contribute to the existing knowledge by suggesting other possible intervention paths through mediating factors. Interventions that improve anxiety, depression, and distress severity may be effective in reducing positive psychotic symptoms among patients with schizophrenia having experienced lifetime trauma. Future experimental research is needed to explore the indirect effects of these interventions on positive psychotic symptoms in patients with schizophrenia.

### Limitations

There are possible limitations to be discussed. The data’s cross-sectional nature limits the ability to pull causal conclusions. There is also a risk of selection bias, given the nature of the sample included in our study, which limits the ability to generalize to the broader array of schizophrenia spectrum disorder patients. Indeed, outpatients and short-stay in-patients with schizophrenia may exhibit different characteristics and should be the subject of future studies. In addition, the sample was relatively small. Further studies with a larger sample are required to better assess the associations in this study. Residual confounding bias is also possible since not all factors associated with positive psychotic symptoms were considered in this study. The use of a self-administered questionnaire and a lack of objective assessment of cognitive functions pose a risk for information bias.

## Conclusion

The findings of this study provide evidence for the association of childhood trauma with positive psychotic symptoms in schizophrenia along with the importance of anxiety, distress, and depression as mediators of this association. Providing a safe environment free from mood dysregulation is the key to reducing the severity of positive psychotic symptoms, and in turn, enhancing the quality of life of patients with schizophrenia. Future investigations with a larger, more diverse sample group and more detailed testing are needed to better understand the relationship between trauma and positive psychotic symptoms in patients with schizophrenia.

## Data Availability

All data generated or analyzed during this study are not publicly available to maintain the privacy of the individuals’ identities. The dataset supporting the conclusions is available upon request to the corresponding author.

## References

[1] Owen MJ, Sawa A, Mortensen PB (2016). Schizophrenia. Lancet.

[2] Abuse S, Administration MHS (2016). Impact of the DSM-IV to DSM-5 changes on the National Survey on drug use and health.

[3] Popovic D, Schmitt A, Kaurani L, Senner F, Papiol S, Malchow B, Fischer A, Schulze TG, Koutsouleris N, Falkai P (2019). Childhood trauma in schizophrenia: current findings and research perspectives. Front Neurosci.

[4] Correll CU, Schooler NR (2020). Negative symptoms in schizophrenia: a review and clinical guide for recognition, assessment, and treatment. Neuropsychiatr Dis Treat.

[5] Schizophrenia [https://www.who.int/news-room/fact-sheets/detail/schizophrenia].

[6] Longden E, Sampson M, Read J (2016). Childhood adversity and psychosis: generalised or specific effects?. Epidemiol Psychiatr Sci.

[7] Van Os J, Kenis G, Rutten BP (2010). The environment and schizophrenia. Nature.

[8] McCutcheon RA, Marques TR, Howes OD (2020). Schizophrenia—an overview. JAMA psychiatry.

[9] Coughlan H, Cannon M (2017). Does childhood trauma play a role in the aetiology of psychosis? A review of recent evidence. BJPsych Advances.

[10] Stanton KJ, Denietolis B, Goodwin BJ, Dvir YJC, Clinics AP (2020). Childhood trauma and psychosis: an updated review. Child Adolesc Psychiatr Clin N Am.

[11] Bailey T, Alvarez-Jimenez M, Garcia-Sanchez AM, Hulbert C, Barlow E, Bendall S (2018). Childhood trauma is associated with severity of hallucinations and delusions in psychotic disorders: a systematic review and Meta-analysis. Schizophr Bull.

[12] Jiang S, Postovit L, Cattaneo A, Binder EB, Aitchison KJ (2019). Epigenetic modifications in stress response genes associated with childhood trauma. Front Psychiatry.

[13] Copeland WE, Shanahan L, Hinesley J, Chan RF, Aberg KA, Fairbank JA, van den Oord EJCG, Costello EJ (2018). Association of childhood trauma exposure with adult psychiatric disorders and functional outcomes. JAMA Netw Ope.

[14] Larsson S, Andreassen OA, Aas M, Røssberg JI, Mork E, Steen NE, Barrett EA, Lagerberg TV, Peleikis D, Agartz IJCP (2013). High prevalence of childhood trauma in patients with schizophrenia spectrum and affective disorder. Compr Psychiatry.

[15] Uyan TT, Baltacioglu M, Hocaoglu C (2022). Relationships between childhood trauma and dissociative, psychotic symptoms in patients with schizophrenia: a case–control study. Gen Psychiatr.

[16] Varese F, Smeets F, Drukker M, Lieverse R, Lataster T, Viechtbauer W, Read J, Van Os J, RP B (2012). Childhood adversities increase the risk of psychosis: a meta-analysis of patient-control, prospective-and cross-sectional cohort studies. Schizophr Bull.

[17] Hany M, Rehman B, Azhar Y, Chapman J (2022). Schizophrenia.

[18] Subica AM, Claypoole KH, Wylie AM (2012). PTSD'S mediation of the relationships between trauma, depression, substance abuse, mental health, and physical health in individuals with severe mental illness: evaluating a comprehensive model. Schizophr Res.

[19] Cotter J, Kaess M, Yung A (2015). Childhood trauma and functional disability in psychosis, bipolar disorder and borderline personality disorder: a review of the literature. Ir J Psychol Med.

[20] Kilcommons A, A M (2005). Relationships between trauma and psychosis: an exploration of cognitive and dissociative factors. Acta Psychiatr Scand.

[21] Gibson LE, Alloy LB, Ellman LM (2016). Trauma and the psychosis spectrum: a review of symptom specificity and explanatory mechanisms. Clin Psychol Rev.

[22] Mandelli L, Petrelli C, Serretti A (2015). The role of specific early trauma in adult depression: a meta-analysis of published literature. Childhood trauma and adult depression. Eur Psychiatry.

[23] Aas M, Henry C, Andreassen OA, Bellivier F, Melle I, Etain B (2016). The role of childhood trauma in bipolar disorders. Int J Bipolar Disord.

[24] Rikani AA, Choudhry Z, Choudhry AM, Ikram H, Asghar MW, Kajal D, Waheed A, Mobassarah NJ (2013). A critique of the literature on etiology of eating disorders. Ann Neurosci.

[25] Bornovalova MA, Huibregtse BM, Hicks BM, Keyes M, McGue M, Iacono W (2013). Tests of a direct effect of childhood abuse on adult borderline personality disorder traits: a longitudinal discordant twin design. J Abnorm Psychol.

[26] Maniglio R (2011). The role of child sexual abuse in the etiology of substance-related disorders. J Addict Dis.

[27] Keen N, Hunter ECM, Peters E (2017). Integrated trauma-focused cognitive-behavioural therapy for post-traumatic stress and psychotic symptoms: a case-series study using imaginal reprocessing strategies. Front Psychiatry.

[28] Morrison AP (2001). The interpretation of intrusions in psychosis: an integrative cognitive approach to hallucinations and delusions. Behavioural and cognitive psychotherapy.

[29] Millan MJ, Agid Y, Brüne M, Bullmore ET, Carter CS, Clayton NS, Connor R, Davis S, Deakin B, RJ DR (2012). Cognitive dysfunction in psychiatric disorders: characteristics, causes and the quest for improved therapy. Nat Rev Drug Discov.

[30] Haddad C, Salameh P, Sacre H, Clément J-P, Calvet B (2021). General description of cognitive deficits in schizophrenia and assessment tools in Lebanon: A scoping review. Schizophr Res Cogn.

[31] Reichenberg A, Velthorst E, Davidson M (2019). Cognitive impairment and psychosis in schizophrenia: independent or linked conditions?. World Psychiatry.

[32] Carrilho CG, Cougo SS, Bombassaro T, Varella AAB, Alves GS, Machado S, Murillo-Rodriguez E, Malaspina D, Nardi AE, Veras AB (2019). Early trauma and cognitive functions of patients with schizophrenia. Front Psychiatry.

[33] Shannon C, Douse K, McCusker C, Feeney L, Barrett S, Mulholland C (2011). The association between childhood trauma and memory functioning in schizophrenia. Schizophr Bull.

[34] Kilian S, Burns J, Seedat S, Asmal L, Chiliza B, Du Plessis S, Olivier M, Kidd M, Emsley R (2017). Factors moderating the relationship between childhood trauma and premorbid adjustment in first-episode schizophrenia. PLoS One.

[35] WtV H, Kreis I, Tjelmeland H, Pfuhl G (2020). Psychosis and psychotic-like symptoms affect cognitive abilities but not motivation in a foraging task. Front Psychol.

[36] Robertson I, Cheung A, Fan X (2019). Insomnia in patients with schizophrenia: current understanding and treatment options. Prog Neuro-Psychopharmacol Biol Psychiatry.

[37] Fekih-Romdhane F, Hallit S, Cheour M, Jahrami H. The nature, consequences, mechanisms, and management of sleep disturbances in individuals at-risk for psychosis. Front Psychiatry. 2022;13:1011963. 10.3389/fpsyt.2022.1011963. 10.3389/fpsyt.2022.1011963 PMC9530454 36203842

[38] Subramaniam M, Abdin E, Shahwan S, Satghare P, Vaingankar JA, Sendren JR, Picco L, Chua BY, Ng BT, Chong SA (2018). Prevalence, correlates and outcomes of insomnia in patients with first episode psychosis from a tertiary psychiatric institution in Singapore. Gen Hosp Psychiatry.

[39] Kaskie RE, Graziano B, Ferrarelli F (2017). Schizophrenia and sleep disorders: links, risks, and management challenges. Nature and science of sleep.

[40] Cosgrave J, Haines R (2018). Van Heugten-van der Kloet D, purple R, Porcheret K, Foster R, Wulff K: the interaction between subclinical psychotic experiences, insomnia and objective measures of sleep. Schizophr Res.

[41] Andorko ND, Millman ZB, Klingaman E, Medoff D, Kline E, DeVylder J, Reeves G, Schiffman J (2018). Association between sleep, childhood trauma and psychosis-like experiences. Schizophr Res.

[42] Seelig AD, Jacobson IG, Donoho CJ, Trone DW, Crum-Cianflone NF, Balkin TJJS (2016). Sleep and health resilience metrics in a large military cohort. Sleep.

[43] Barlati S, Nibbio G, Vita A (2021). Schizophrenia during the COVID-19 pandemic. Curr Opin Psychiatry.

[44] Zhand N, Joober R. Implications of the COVID-19 pandemic for patients with schizophrenia spectrum disorders: narrative review. BJPsych Open. 2021;7(1):e35. 10.1192/bjo.2020.157. 10.1192/bjo.2020.157 PMC7804069 33431109

[45] Kozloff N, Mulsant BH, Stergiopoulos V, Voineskos AN (2020). The COVID-19 global pandemic: implications for people with schizophrenia and related disorders. Schizophr Bull.

[46] Parra A, Juanes A, Losada CP, Álvarez-Sesmero S, Santana VD, Martí I, Urricelqui J, Rentero D (2020). Psychotic symptoms in COVID-19 patients. A retrospective descriptive study. Psychiatry Res.

[47] Tsur N, Abu-Raiya H (2020). COVID-19-related fear and stress among individuals who experienced child abuse: the mediating effect of complex posttraumatic stress disorder. Child Abuse Negl.

[48] Negele A, Kaufhold J, Kallenbach L, Leuzinger-Bohleber M (2015). Childhood trauma and its relation to chronic depression in adulthood. Depress Res Treat.

[49] Kascakova N, Furstova J, Hasto J, Madarasova Geckova A, Tavel P (2020). The unholy trinity: childhood trauma, adulthood anxiety, and long-term pain. Int J Environ Res Public Health.

[50] Kuzminskaite E, Penninx BWJH, van Harmelen A-L, Elzinga BM, Hovens JGFM, Vinkers CH (2021). Childhood trauma in adult depressive and anxiety disorders: an integrated review on psychological and biological mechanisms in the NESDA cohort. J Affect Disord.

[51] Hartley S, Barrowclough C, Haddock G (2013). Anxiety and depression in psychosis: a systematic review of associations with positive psychotic symptoms. Acta Psychiatr Scand.

[52] Naidu K, van Staden WC, van der Linde M (2014). Severity of psychotic episodes in predicting concurrent depressive and anxiety features in acute phase schizophrenia. BMC Psychiatry.

[53] Wigman JT, van Nierop M, Vollebergh WA, Lieb R, Beesdo-Baum K, Wittchen H-U, van Os J (2012). Evidence that psychotic symptoms are prevalent in disorders of anxiety and depression, impacting on illness onset, risk, and severity—implications for diagnosis and ultra–high risk research. Schizophr Bull.

[54] Boyer L, Millier A, Perthame E, Aballea S, Auquier P, Toumi M (2013). Quality of life is predictive of relapse in schizophrenia. BMC Psychiatry.

[55] Viswanath B, Chaturvedi SK (2012). Cultural aspects of major mental disorders: a critical review from an Indian perspective. Indian J Psychol Med.

[56] Gerges S, Haddad C, Daoud T, Tarabay C, Kossaify M, Haddad G, Hallit S (2022). A cross-sectional study of current and lifetime sexual hallucinations and delusions in Lebanese patients with schizophrenia: frequency, characterization, and association with childhood traumatic experiences and disease severity. BMC Psychiatry.

[57] Fekih-Romdhane F, Abboud C, Kossaify M, El Khoury N, Sleiman YB, Hachem D, Haddad G, Hallit S (2022). Child abuse and aggressiveness in individuals diagnosed with schizophrenia in Lebanon. Perspect Psychiatr Care.

[58] Mørkved N, Johnsen E, Kroken RA, Gjestad R, Winje D, Thimm J, et al. Does childhood trauma influence cognitive functioning in schizophrenia? The association of childhood trauma and cognition in schizophrenia spectrum disorders. Schizophr Res Cogn. 2020;21:100179. 10.1016/j.scog.2020.100179. 10.1016/j.scog.2020.100179 PMC7240182 32461919

[59] Alkhunizan M, Alkhenizan A, Basudan L (2018). Prevalence of mild cognitive impairment and dementia in Saudi Arabia: a community-based study. Dementia and Geriatric Cognitive Disorders EXTRA.

[60] Saleh AA, Alkholy RSAEHA, Khalaf OO, Sabry NA, Amer H, El-Jaafary S, Khalil MAEF (2019). Validation of Montreal cognitive assessment-basic in a sample of elderly Egyptians with neurocognitive disorders. Aging Ment Health.

[61] Freud T, Vostrikov A, Dwolatzky T, Punchik B, Press Y (2020). Validation of the Russian version of the MoCA test as a cognitive screening instrument in cognitively asymptomatic older individuals and those with mild cognitive impairment. Frontiers in Medicine.

[62] Merza K, Papp G, Molnár J, Kuritárné Szabó I (2017). Characteristics and development of nonsuicidal super self-injury among borderline inpatients. Psychiatr Danub.

[63] Park K, Shim G, Jeong B (2020). Validation of the Traumatic Antecedents Questionnaire using item response theory. Brain Behav.

[64] First M, Spitzer R, Gibbon M, Williams JJSB (2002). Structured clinical interview for DSM-IV-TR Axis I disorders, research version, Patient Edition (SCID-I/P).

[65] Hallit S, Obeid S, Haddad C, Kazour F, Kazour G (2017). Validation of the Arabic Version of the PANSS scale among Lebanese schizophrenic patients. J Psychopathol.

[66] Hallit S, Obeid S, Haddad C, Hallit R, Akel M, Haddad G, Soufia M, Khansa W, Khoury R, Kheir N (2020). Construction of the Lebanese anxiety scale (LAS-10): a new scale to assess anxiety in adult patients. Int J Psychiatry Clin Pract.

[67] Merhy G, Azzi V, Salameh P, Obeid S, Hallit S (2021). Anxiety among Lebanese adolescents: scale validation and correlates. BMC Pediatr.

[68] Hallit S, Sacre H, Haddad C, Malaeb D, Al Karaki G, Kheir N, Hajj A, Hallit R, Salameh P (2019). Development of the Lebanese insomnia scale (LIS-18): a new scale to assess insomnia in adult patients. BMC Psychiatry.

[69] Obeid S, Hallit CAE, Haddad C, Hany Z, Hallit S (2018). Validation of the Hamilton Depression Rating Scale (HDRS) and sociodemographic factors associated with Lebanese depressed patients. Encephale.

[70] Malaeb D, Farchakh Y, Haddad C, Sacre H, Obeid S, Hallit S, Salameh P (2022). Validation of the Beirut Distress Scale (BDS-10), a short version of BDS-22, to assess psychological distress among the Lebanese population. Perspect Psychiatr Care.

[71] Alyami M, Henning M, Krägeloh CU, Alyami H (2021). Psychometric evaluation of the Arabic version of the Fear of COVID-19 Scale. Int J Ment Health Addict.

[72] Ahorsu DK, Lin CY, Imani V, Saffari M, Griffiths MD, Pakpour AH (2022). The fear of COVID-19 scale: development and initial validation. Int J Ment Health Addict.

[73] Hayes AF. Introduction to mediation, moderation, and conditional process analysis: a regression-based approach. Guilford publications; 2017.

[74] Upthegrove R, Marwaha S, Birchwood M (2017). Depression and schizophrenia: cause, consequence, or trans-diagnostic issue?. Schizophr Bull.

[75] Ruby E, Rothman K, Corcoran C, Goetz RR, Malaspina D (2017). Influence of early trauma on features of schizophrenia. Early Interv Psychiatry.

[76] Xie P, Wu K, Zheng Y, Guo Y, Yang Y, He J, Ding Y, Peng HJJoad: , Prevalence of childhood trauma and correlations between childhood trauma, suicidal ideation, and social support in patients with depression, bipolar disorder, and schizophrenia in southern China, J Affect Disord. 2018, 228:41-48. 10.1016/j.jad.2017.11.011 29223913

[77] Bebbington P, Jonas S, Kuipers E, King M, Cooper C, Brugha T, Meltzer H, McManus S, Jenkins R (2011). Childhood sexual abuse and psychosis: data from a cross-sectional national psychiatric survey in England. Br J Psychiatry.

[78] Harley M, Kelleher I, Clarke M, Lynch F, Arseneault L, Connor D, Fitzpatrick C, Cannon M (2010). Cannabis use and childhood trauma interact additively to increase the risk of psychotic symptoms in adolescence. Psychol Med.

[79] Houston J, Murphy J, Shevlin M, Adamson GJPM (2011). Cannabis use and psychosis: re-visiting the role of childhood trauma. Psychol Med.

[80] Myin-Germeys I, van Os J (2007). Stress-reactivity in psychosis: evidence for an affective pathway to psychosis. Clin Psychol Rev.

[81] van Nierop M, Viechtbauer W, Gunther N, Van Zelst C, De Graaf R, Ten Have M, Van Dorsselaer S, Bak M, Risk G, van Winkel R (2015). Childhood trauma is associated with a specific admixture of affective, anxiety, and psychosis symptoms cutting across traditional diagnostic boundaries. Psychol Med.

[82] Bloomfield MAP, Chang T, Woodl MJ, Lyons LM, Cheng Z, Bauer-Staeb C, Hobbs C, Bracke S, Kennerley H, Isham L (2021). Psychological processes mediating the association between developmental trauma and specific psychotic symptoms in adults: a systematic review and meta-analysis. World psychiatry : official journal of the World Psychiatric Association (WPA).

[83] Singh GK, Kenney MK. Rising prevalence and neighborhood, social, and behavioral determinants of sleep problems in US children and adolescents, 2003-2012. Sleep Disord. 2013;2013:394320. 10.1155/2013/394320. 10.1155/2013/394320 PMC3683488 23819057

[84] Thompson A, Lereya S, Lewis G, Zammit S, Fisher H, Wolke D (2015). Childhood sleep disturbance and risk of psychotic experiences at 18: UK birth cohort. Br J Psychiatry.

[85] Finatti F, Pigato G, Pavan C, Toffanin T, Favaro A (2020). Psychosis in patients in COVID-19-related quarantine: a case series. Prim Care Companion CNS Disord.

[86] Trnka R, Lorencova R (2020). Fear, anger, and media-induced trauma during the outbreak of COVID-19 in the Czech Republic. Psychol Trauma.

[87] van den Berg DP, de Bont PA, van der Vleugel BM, de Roos C, de Jongh A, Van Minnen A, van der Gaag M (2015). Prolonged exposure vs eye movement desensitization and reprocessing vs waiting list for posttraumatic stress disorder in patients with a psychotic disorder: a randomized clinical trial. JAMA Psychiatry.

[88] Peters E, Ward T, Jackson M, Morgan C, Charalambides M, McGuire P, Woodruff P, Jacobsen P, Chadwick P, Garety PAJWP (2016). Clinical, socio-demographic and psychological characteristics in individuals with persistent psychotic experiences with and without a “need for care”. World Psychiatry.

[89] Cragin CA, Straus MB, Blacker D, Tully LM, Niendam TA (2017). Early psychosis and trauma-related disorders: clinical practice guidelines and future directions. Frontiers in psychiatry.

